# Knowledge about HIV prevention and transmission among recently diagnosed tuberculosis patients: a cross sectional study

**DOI:** 10.1186/1471-2458-13-1237

**Published:** 2013-12-28

**Authors:** Cesar Ugarte-Gil, Mario Ponce, Carlos Zamudio, Luz Canaza, Frine Samalvides, Carlos Seas

**Affiliations:** 1 Instituto de Medicina Tropical Alexander Von Humboldt, Universidad Peruana Cayetano Heredia, Av. Honorio Delgado 430 SMP, Lima31, Lima, Peru; 2 Departamento de Enfermedaded Infecciosas, Tropicales y Dermatológicas, Hospital Nacional Cayetano Heredia, Lima, Peru

**Keywords:** HIV, Tuberculosis, Knowledge, Prevention

## Abstract

**Background:**

Patients with Tuberculosis (TB) are a vulnerable group for acquiring HIV infection. Therefore, countries with a concentrated HIV epidemic and high prevalence of TB should provide adequate information about HIV prevention to TB patients.

**Methods:**

We conducted a cross-sectional study to evaluate the level of knowledge on HIV prevention and transmission among newly diagnosed TB patients in Lima, Peru. The survey evaluated knowledge about HIV infection and prevention and was administered before HIV counseling and blood sampling for HIV testing were performed.

**Results:**

A total of 171 TB patients were enrolled; mean age was 31.1 years, 101 (59%) were male. The overall mean level of knowledge of HIV was 59%; but the specific mean level of knowledge on HIV transmission and prevention was only 33.3% and 41.5%, respectively. Age and level of education correlated with overall level of knowledge in the multivariate model (P-value: 0.02 and <0.001 respectively).

**Conclusions:**

The study shows inadequate levels of knowledge about HIV transmission and prevention among newly-diagnosed TB patients in this setting, and underscores the need for implementing educational interventions in this population.

## Background

In 2011, the global number of new tuberculosis (TB) cases was 8.7 million; 13% of these cases were co-infected with the human immune deficiency virus (HIV), and only 40% of TB notified cases were tested for HIV [1]. Peru reported an incidence of pulmonary TB of 101 cases per 100 000 habitants in 2011, based on the WHO Global Tuberculosis Control report of 2012 [1]. Based on the same report, only 21% of all Peruvian TB patients knew their HIV status (14% were HIV positive) [1]. TB is specially concentrated in urban areas in Peru, where the capital city, Lima, houses 58% of TB cases [2]. The HIV situation in Peru is that of a concentrated epidemic [3]. Lima reports the highest incidence rate of HIV infection in the country [4,5], creating a favorable scenario for the co-infection of HIV and TB. In fact, since the beginning of the HIV epidemic in Peru, TB has been recognized as a significant cause of morbidity and mortality among HIV-infected patients [6,7].

An essential component of the Global Plan to Stop TB 2011–2015 is to empower TB patients, pursuing advocacy, communication, and community participation in health care and prevention [8]. Integration of TB and HIV activities at the community health level is also essential to reach the pre-specified goals for 2015. To implement HIV prevention strategies among TB patients it is essential to know the level of knowledge about HIV in this specific population. Several studies [9-12] have evaluated the level of HIV knowledge in different Peruvian populations, and some of these studies have addressed vulnerable populations [10], but there is no information available on this topic in TB patients in Peru.

We conducted this study to evaluate the level of knowledge about HIV among newly diagnosed TB patients in the district of San Juan de Lurigancho, the most densely populated district of Peru with a population of approximately 1 million inhabitants living within a surface area of 131.21 km^2^. In 2010, this district of Lima reported incidences of TB and HIV of 193 and 26.7 per 100 000 inhabitants, respectively [13].

## Methods

### Study population

We conducted a cross-sectional study, from October 2010 to November 2011, among adult patients recently diagnosed with pulmonary TB, confirmed by microscopy and/or culture, with no previous diagnosis of HIV infection, at peripheral health care centres of San Juan de Lurigancho district in Lima, Peru. There are over 30 peripheral health care centres in this district; every centre has an office from the National TB Program (NTP) to provide directly observed therapy (DOT).

### Questionnaire

The instrument to measure knowledge about HIV was a self-administered questionnaire containing 11 questions about socio-demographic information, TB history, previous HIV tests and 17 multiple-choice questions about knowledge of HIV infection, transmission and prevention. The maximum score for HIV knowledge was 35 points, reflecting 100% overall level of knowledge. This instrument has been applied before in a similar population in Lima [9], but was validated again with a pilot study before using it in this study population (Chronbach’s alpha (0.6)).

### Data analysis

Since HIV counseling is mandatory before testing, the questionnaire was applied before counseling and blood sampling for HIV testing were performed. Considering a confidence level of 95%, a power of 80% and a level of knowledge of 60-70% (based on previous studies) [9-12], the minimum sample size was 171 participants. To reach a better representative sample, the sample size by each center was weighted by the number of TB patients seen by these centers in 2009. A convenience sampling was used until each center reached its sample size.

Data were entered in Microsoft Access 2007 database (Microsoft, US) and was analysed using STATA v12.0 (StataCorp, US). Mean differences in the level of knowledge across demographic groups, socio-economic groups and level of education were evaluated. A backward stepwise linear regression model was used to evaluate the association between level of knowledge (percentage of correct answers on the questionnaire) and predictor variables. A 0.05 level of significance was used throughout.

### Ethical considerations

All participants gave signed consent to participate in the study. The Institutional Review Board from Universidad Peruana Cayetano Heredia and the Health Direction of San Juan de Lurigancho district approved the study.

## Results

A total of 171 participants were interviewed. The mean age was 31.1 years (median age was 26), 101(59%) were males, 15 (8.8%) had a previous TB episode and 44 (25.7%) had a previous HIV test (all negative). Males had more sexual partners and a higher proportion of reported use of condoms compared with females. Other characteristics of the participants are presented in Table 1.

**Table 1:** Characteristics of the study population

**Characteristics**	**Total (n = 171)**	**Male (n = 101)**	**Female (n = 70)**	P- value**
**Mean age (SD)**	31.1 (13)	30.7 (13.2)	31.5 (12.8)	0.7
**Marital status**				
***Single (%)***	104 (60.8%)	69 (68.3%)	35 (70%)	0.8
**Level of education**				
***Primary school (%)***	23 (13.5%)	14 (13.9%)	9 (12.9%)	0.9
***Secondary school (%)***	103 (60.2%)	66 (65.4%)	37 (52.9%)	0.2
***Above secondary school (%)***	45 (26.3%)	21 (20.8%)	24 (34.3%)	0.3
**Previous TB episode (%)**	15 (8.8%)	4 (5.7%)	11 (10.9%)	0.2
**Median number of sexual partners (Range)**	1 (0–21)	2 (0–21)	1 (0–10)	<0.001
**Reported use of condoms (%)**	70 (40.9%)	36 (35.6%)	19 (27.1%)	0.2
Mean level of knowledge, % (SD)*	59 (15.7)	57.4 (15.7)	61.3 (15.5)	0.11

*Highest possible level of knowledge is 100% = 35 points.

**Comparison between males and females; t-test for difference of means, Man-Whitney for medians and chi-square for difference in proportions.

The mean percent correct answers regarding knowledge on HIV was 59% (SD: 15.7). None of the participants obtained the 100% level of knowledge (min: 22.9%; max: 97.1%). Only 33.3% and 41.5% had correct answers regarding knowledge of ways for HIV transmission and knowledge of preventive practices, respectively. A total of 83 participants (48.5%) considered that the condom should be used always for vaginal, anal and oral sex, but only 55 participants (32.2%) reported regular condom use.

The overall level of knowledge increased by education level: 45.2% for participants who completed primary school level, 57.8% for participants who completed secondary school level, and 68.6% for participants with greater than secondary school education. Women had a higher level of knowledge (mean: 61.3%; SD: 15.5) compared with men (mean: 57.4%; SD: 15.7).

The univariate analysis is presented in Table 2. Only education level and age showed significant association with overall level of knowledge. Mean level of knowledge was associated positively with education, with an increase of 11.6 percentage points (95% CI: 8.1- 15.0; p-value < 0.001) by each level of education. However, it was associated negatively with age in the group older than 26 years, decreasing by 1.33 percentage points per year of age (95% CI: -2.43 - -0.22; p-value = 0.02). Age younger than 26 years old, gender, single status, previous TB episode and number of sexual partners did not show statistically significant correlation with level of knowledge. The multivariate regression model is shown in Table 3 and included age older than 26 years and level of education, showing a decrease in the level of knowledge with older age (0.25 percentage points mean decrease per 1 year increase in age, p-value = 0.01), and an increase in the mean level of knowledge with higher level of education (mean increase 10.6 percentage points, p-value < 0.001).

**Table 2:** Univariate regression model

**Variables**	**Regression coefficient**	**95% CI**	**P-value**
Age < 26 years old	0.85	-0.1 – 1.8	0.08
Age > 26 years old	-1.32	-2.4 – –0.2	0.02
Single	-1.1	-3.1 – 0.9	0.3
Male	-3.9	-8.7 – 0.9	0.1
Previous TB episode	1.13	-7.3 – 9.5	0.8
Number of sexual partners	-0.35	-1.3 – 0.5	0.4
Education	11.5	8.1 – 15	<0.001

**Table 3:** Multivariate regression model

**Variables**	**Regression coefficient**	**95% CI**	**P-value**
Age > 26 years old	-0.25	-0.44 – –0.06	0.01
Education	10.63	7.2 – 14.1	<0.001

## Discussion

This study found a low level of knowledge about HIV transmission and prevention among newly diagnosed TB patients in San Juan de Lurigancho. This main finding of the study, taken into account along with the low percentage of regular condom use in TB patients puts this population at risk to acquire HIV and other sexually transmitted diseases.

In addition, two important findings in our study should be pointed out. First, we observed a decrease in the level of knowledge on HIV transmission and prevention by age. This finding can be explained by a “cohort effect” [14]. Younger participants had more access to and had higher chances of receiving education on this topic compared with older participants. At the beginning of the HIV epidemic in the 1980s and early 1990s, information about HIV prevention was not widely available for all, therefore, youth at that time were unlikely to be exposed to adequate information for HIV prevention. However, despite more opportunities to exposure to HIV prevention information among younger populations in Peru, the reported median age of HIV infection is around 20 years old in Peru [15]. Second, an increase in the level of knowledge was observed with level of education in this study. A systematic review showed that most educated people had a lower risk of HIV infection. This review found that the HIV prevalence dropped more consistently in this group compared with less educated people [16].

Some studies have shown the importance of offering HIV counseling to TB patients [16,17]. HIV testing was offered to all TB patients in the study area for free. However, at a national level, the percentage of TB patients with known HIV status has been decreasing over recent years (35%, 29% and 21% for 2009, 2010 and 2011, respectively) [1]. The NTP provides regular preventing activities for TB among TB patients and their relatives, but it is necessary to implement more collaborative activities between the NTP and HIV programs, to improve the detection and prevention of HIV infection among TB patients at a national level. WHO remarks the importance of the integration of both programs in prevention and treatment [18], especially in settings with limited resources. For example, experiences in Malawi and South Africa showed that local integrated programs could operate successfully [19]. One systematic review showed that the referral model (where TB patients are referred to HIV service for HIV diagnosis, and vice versa) could be the less complex model, but still have the risk of failure in the referral process (the patient does not attend the appointment for several factors) [20]. The referral model is currently in place in Peru. The lower cost of this model compared to a fully-integrated model is a potential advantage, but it has to be weighted against the big weakness of losing referrals (only 21% of TB patients know their HIV status in Peru despite of free access). There is also a lack of information of programmatic and logistic issues of integrated TB-HIV services in settings without high HIV prevalence such as Peru (most of the studies are based in African countries) [21-23]. As the evidence shows, the integration of these programs cannot be performed with a unique recipe for all settings.

The importance of HIV testing and counseling is not only to promptly identify HIV infected patients, but also provides the opportunity to talk about HIV prevention [24]. In the case of TB patients in Peru, because they are a generally young population and share similar demographic characteristics with vulnerable populations for HIV infection, HIV counseling and testing activities at the time of TB testing can provide the opportunity to address the potential lack of HIV knowledge among these vulnerable populations.

Some public health programs (such as NTP or pregnancy program) in Peru provide free HIV counseling and free HIV testing to benefit their patients as described before. In recent years, the Peruvian Government, non-governmental organizations, academic institutions, and international initiatives, such as the Global Fund have developed activities to provide information about HIV prevention to different populations in the country [14]. As a result of these and other initiatives, the level of knowledge on HIV prevention among women increased from 18.9% in 2000 to 68.2% in 2011, based on information from the National Survey for Demography and Family Health (ENDES 2011) [25].

Other studies have shown a similar level of knowledge as in our study among people seeking HIV counseling in a referral hospital in Lima [9]. However, a comparison of these studies with the information provided in our study is difficult to make. Reasons for that include different questionnaires used to evaluate level of knowledge and no detailed evaluation of factors associated with the level of knowledge in these studies [10-12,25]. At a more global level, there is a lack of information about the level of HIV knowledge among TB patients. One study in China reported only 1.7% level of knowledge about HIV among 2300 TB patients [26], and another study in Afghanistan showed that 23.3% of tuberculosis patients knew about HIV [27]. No studies on this topic are available from Latin America.

This study has several limitations; one of these is selection bias. Efforts were made to minimize this bias using intensive enrolment of study subjects during TB clinic attention hours. In addition, HIV carries a stigma in our population, and some of the questions, especially the questions regarding sexual practices, may be affected by information bias (specifically recall bias). Another limitation of this and other similar studies is the lack of a standardized tool for evaluating level of HIV knowledge, which makes comparison across studies difficult.

## Conclusion

After more than 25 years since the first case of HIV in Peru, there is still lack of knowledge on HIV prevention in the TB infected population. Although Peru does not have an HIV/TB problem of the same magnitude as Sub-Saharan African countries, our results indicate that it is necessary to put more efforts towards HIV prevention among this population. Specific interventions directed at TB patients, implemented in coordination between HIV and TB programs, are necessary to address this issue.

## Competing interests

We declare that we do not have competing interests.

## Authors’ contributions

CUG, MP, CZ, FS and CS participated in the conception and design of the study. CUG coordinated the data collection and CUG, MP and LC involved in the fieldwork. CUG, MP and CZ analyzed the data; CUG drafted the manuscript. CS, CZ, FS, MP edited the manuscript. All authors read and approved the final manuscript.

## Pre-publication history

The pre-publication history for this paper can be accessed here:

http://www.biomedcentral.com/1471-2458/13/1237/prepub
